# Coping Mechanisms for Lymphedema: An Analysis of Patient Experiences

**DOI:** 10.7759/cureus.41573

**Published:** 2023-07-08

**Authors:** Claudia Admoun, Harvey N Mayrovitz

**Affiliations:** 1 Pediatrics, The University of New Mexico School of Medicine, Albuquerque, USA; 2 Medical Education, Nova Southeastern University Dr. Kiran C. Patel College of Allopathic Medicine, Davie, USA

**Keywords:** compression, lifestyle, diet, exercise, coping mechanisms, lower extremity, upper extremity, bilateral, unilateral, lymphedema

## Abstract

Background and objective

Lymphedema is a condition caused by impaired lymphatic function. Acquired lymphedema is often due to neoplasia, infection, trauma, or radiation damage. Most patients rely on advice from fellow patients and personal research to manage their symptoms. We believe coping mechanisms for lymphedema can be more streamlined and made easily accessible if the most common effective strategies are determined and collected in a single repository for reference. To that end, we aimed to assess the experiences of lymphedema patients and the coping mechanisms they employed.

Methods

Feedback from lymphedema patients was obtained using a 19-item questionnaire-based survey distributed to multiple online lymphedema support groups. It focused on the type of lymphedema and its effect on the respondents and sought data to characterize coping mechanisms that individuals with lymphedema use and their effectiveness.

Results

The respondents (n=400) had a median age of 55 years (range: 18-83 years). The overall mean BMI was 35.8 kg/m^2^, with respondents with lower extremity (LE) lymphedema having a greater mean BMI (38.4 vs. 31.0, p<0.05). Most of the respondents were female (n=382, 95.5%). LE lymphedema was more common (n=280, 70%) than the upper extremity (UE) variant (n=120, 30%). Within the LE group, 99 were unilateral (35.4%) and 181 were bilateral (64.6%). Moderate restriction was the most reported level with 44% in the unilateral group and 64% in the bilateral group. Stretching, low-impact exercise, manual lymphatic drainage (MLD), and compression bandages were the most commonly used coping methods and most of the respondents rated them as somewhat effective. Of note, 30% of respondents in either group rated increased water intake as either slightly, moderately, or very helpful; 25% of respondents also rated decreased alcohol intake as very helpful. The use of a therapist and family and doctor support was rated as extremely helpful. Within the UE group, 105 were unilateral (87.5%) and 15 were bilateral (12.5%). The most common cause of UE lymphedema was breast cancer-related (98/120, 81.7%) followed by melanoma treatment. Mild restriction was the most reported level (n=48, 45.7%). The UE group had similar results as the LE group in terms of coping mechanisms, dietary changes, and psychosocial support.

Conclusion

Based on the experiences of the lymphedema patients surveyed, the management of the condition is multifactorial and hence not compatible with a one-size-fits-all strategy. LE lymphedema was more common than the UE variant; but both groups reported engaging in stretching, low-impact exercise, manual lymphatic drainage, and compression bandages with similar rates of satisfaction reported in both groups. Dietary changes were not commonly employed. Therapy, doctor, and family support were the most commonly used support mechanisms, with high satisfaction among both groups. The overall coping mechanisms and their ratings in terms of efficacy between UE and LE groups were similar although the impact of quality of life was greater for bilateral conditions. We believe our findings represent the first steps to providing information potentially useful to aid future and current lymphedema patients in finding the coping methods that work best for them.

## Introduction

Lymphedema occurs due to impaired lymphatic system function proving inadequate to remove enough interstitial fluids to prevent their abnormal accumulation. One of the major causes of this condition is an injury to or destruction of lymphatic pathways or function resulting from treatment for breast cancer or gynecological cancers, leading to upper extremity (UE) or lower extremity (LE) lymphedema respectively [1]. Chronic pain and heaviness in the affected limb are commonly reported in lymphedema patients [1]. Studies have shown statistically significant poorer social well-being in lymphedema patients, specifically with regard to body image, appearance, and sexuality. Negative social impacts have also been reported, including financial burden, social isolation, and public insensitivity [2]. Lymphedema can lead to life-changing effects among patients, and they may require strategies to optimally cope with the lymphedema and its effects on various aspects of daily life, especially since the disease is not generally curable. The goal of this study was to assess and characterize these coping mechanisms as expressed by persons living with lymphedema. We hope to create a repository of data and information on coping mechanisms that patients could rely on to improve their knowledge base and act as an evidence-based management resource.

## Materials and methods

Participants

Data presented in this study were obtained via an anonymous survey among respondents, which was approved by the Nova Southeastern University Institutional Review Board (IRB No.: 2021-574-NSU). It was posted on the Facebook pages of several lymphedema support groups during the period from March to September 2022. The survey was closed once we received a total of 400 complete responses. The distribution of respondents is shown in Table 1.

**Table 1:** Lymphedema type among respondents

Lymphedema type (N=400)	
Upper extremity lymphedema (n=120)	Unilateral (n=105)
	Bilateral (n=15)
Lower extremity lymphedema (n=280)	Unilateral (n=99)
	Bilateral (n=181)

Survey questions

The survey had 19 questions. Apart from questions on the year of the diagnosis and the respondent’s sex, age, height, and weight, the survey also included 13 questions designed to obtain data about the type of lymphedema and its effect on the respondent and to elicit data to characterize coping mechanisms that persons with lymphedema use and their effectiveness. These questions and answer choices are shown in Table 2.

**Table 2:** Survey questions and answer options A total of 13 questions were included in the survey.

Survey question	Possible respondent selections
What was the cause of your lymphedema?	Breast cancer treatment
	Melanoma treatment
	Gynecologic cancer treatment
	Head and neck cancer treatment
	Prostate cancer treatment
	Lymphoma
	Filariasis
	Infection
	Trauma
	Unknown
	Other
To what degree does your lymphedema condition restrict your daily activities?	No restriction
	Mild
	Moderate
	Severe
	Very severe
What is part of your daily self-care program and what is its effectiveness? Choices: not used, not effective, somewhat effective, very effective	Physical exercise
	Deep breathing techniques
	Compression garments
	Compression bandages
	Sequential pneumatic compression
	Self-manual lymphatic drainage techniques
	Limb elevation
How frequently do you do any of the following as part of your skincare? Choices: never, rarely, sometimes, frequently	Keep the skin of the affected limb extremely clean
	Keep skin hydrated by moisturizing with lotion
	Use natural bug repellant
	Use an electric razor for hair removal
	Avoid excessive nail treatment
	Avoid puncture due to injection or blood draws from the affected limb
	Avoid excessive use of antibiotic ointment for skin wound care
	Avoid excessive sun exposure
	Frequent use of sunscreen when exposed to the sun
What dietary changes have you tried and found helpful in managing your lymphedema? Choices: not tried, tried but not helpful, slightly helpful, moderately helpful, very helpful	Decrease/cessation of caffeine consumption
	High-fiber diet
	High-protein diet
	Low-fat diet
	Low-salt diet
Did you regularly perform any of the following exercises after your lymphedema diagnosis?	Low-impact aerobic exercise (swimming, biking, walking)
	High-impact aerobic exercise (running, jumping)
	Weightlifting with slow gradual progression
	Stretching
	Balance training
	Flexibility training
When traveling on an airplane, do you do any of the following?	Avoid dehydration
	Frequently exercise leg muscles
	Walk in the aisle when possible
	Use limb compression in flight
	Choose the aisle seat for an easier ambulation opportunity
	Minimize the weight of luggage
	None of the above
	Do not travel on the airplane
Starting from three months after your first signs of lymphedema, have you experienced pain?	Yes
	No
If you have experienced pain, what was the severity of your pain?	No pain
	Mild
	Moderate
	Severe
	Very Severe
	Worst pain possible
Regarding your social well-being in terms of body image and appearance, is it true or false that you use the following coping mechanism? Choices: true, false	Lymphedema did not impact my body image
	I underwent cognitive behavioral therapy treatment
	I avoid allowing negative thoughts about my body image
	I avoid comparing myself to others
	I avoid social media impacting my self-image
	I cover up my lymphedema with loose clothes
	I accept my self-image
What are your sources of support regarding your lymphedema condition?	Family
	Friends
	Doctor and healthcare team
	Lymphedema therapist
	On-site lymphedema support group
	Online lymphedema support group
Do you avoid exposing your lymphedema limb to any of the following? Choices: do not avoid, somewhat avoid, frequently avoid	Lifting heavy items
	Wearing a bag with a shoulder strap
	Wearing tight clothes, shoes, or jewelry
	Using a blood pressure cuff
	Crossing leg when sitting
	Immobility for prolonged periods of time
	Sitting in the same position for a prolonged length of time
	Extreme heat
	Extreme cold
If you had undergone surgical treatment for your lymphedema condition, was it helpful in alleviating your symptoms? Choices: did not undergo, not helpful, somewhat helpful, very helpful	Lymph node transplantation
	Lymphovenous bypass
	Liposuction
	Charles procedure (skin grafts)

Data analysis

Data were stratified by the type of lymphedema the respondent had (UE, LE, and whether unilateral or bilateral). Patients with UE unilateral lymphedema and bilateral lymphedema were designated as UELE(1) and UELE(2) respectively. Patients with LE unilateral lymphedema and bilateral lymphedema were designated as LELE(1) and LELE(2) respectively. Statistical analyses were done using IBM SPSS Statistics version 16 (IBM Corp., Armonk, NY). Differences in continuous variables [age, body mass index (BMI), etc.] were tested using independent t-tests. Chi-square tests were used to determine if there was a statistically significant difference between various responses. The parameters assessed included respondents’ lymphedema type, age, gender, BMI, cause of lymphedema, restrictions on everyday life, daily self-care routine, chronic pain, support system, and treatment methods. A p-value <0.05 was taken to indicate a statistically significant difference. Numeric data is presented as mean ± standard deviation (SD).

## Results

Respondent information

As shown in Table 1, there were 400 respondents, and their demographics are summarized in Table 3. Most of them were female (n=382, 95.5%). LE lymphedema was more common (n=280) than the UE variant (n=120). The mean age for the entire cohort was 55 ± 12 years, and there was no significant difference between the groups in terms of age (56 ± 11 vs. 55 ± 13). The overall BMI was 35.8 kg/m^2^, with respondents with LE lymphedema having a statistically significant higher BMI (38.4 vs. 31.0, p<0.05) compared to their UE counterparts. The most common cause of UE lymphedema was breast cancer-related (98/120, 81.7%) followed by melanoma treatment. The most common cause in the LE group was reported as unknown (n=100/280, 35.7%) followed by "other" (82/280, 29.2%).

**Table 3:** Respondent information (N=400) SD: standard deviation

Characteristics		
Lymphedema limb	Upper extremity (n=120)	Lower extremity (n=280)
Age, years, mean ± SD (overall: 55 ± 12)	56 ± 11	55 ± 13
BMI, kg/m^2^, mean (overall: 35.8)	31	38.4
Unilateral lymphedema, n (%)	105 (87.5)	99 (35.4)
Bilateral lymphedema, n (%)	15 (12.5)	181 (64.6)
Etiology of lymphedema, n (%)		
Breast cancer	98 (82)	3 (1.1)
Melanoma treatment	7 (5.8)	11 (3.9)
Gynecologic cancer	2 (1.6)	47 (16.7)
Infection	1 (0.9)	15 (5.3)
Trauma	3 (2.5)	19 (6.7)
Head and neck cancer	1 (0.9)	2 (0.7)
Lymphoma	0, (0)	1 (0.3)
Unknown	5 (4.2)	100 (35.7)
Other	3 (2.5)	82 (29.2)

Patients with upper extremity lymphedema

As noted in Table 3, there were 120 patients with UE lymphedema

Restrictions on Activities of Daily Living

As shown in Table 4, 41% of patients who had UELE(1) reported mild restrictions and 32% reported moderate restrictions. Only 11% of those with unilateral lymphedema reported restrictions to be severe or very severe whereas 32% of patients with UELE(2) reported severe or very severe restrictions.

**Table 4:** Reported restriction levels due to upper extremity lymphedematous condition

	Unilateral, %	Bilateral, %
No restriction	14.3	6.7
Mild	41.0	33.3
Moderate	32.4	26.7
Severe	9.5	26.7
Very severe	2.9	6.7

Exercise Methods Employed and Their Reported Effectiveness

**Figure 1:**
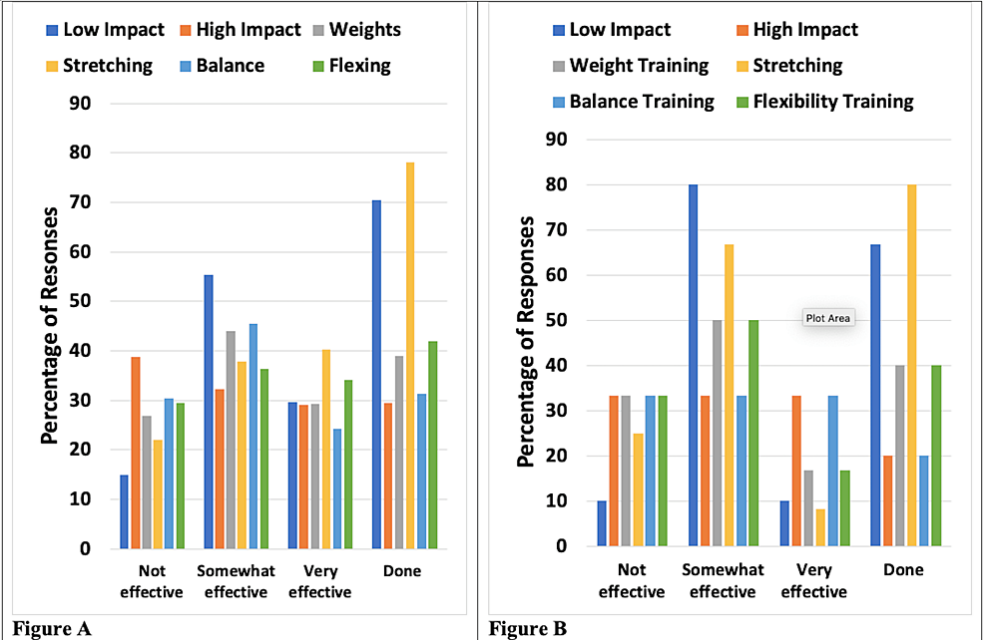
Exercise types and their reported effectiveness for patients with upper extremity lymphedema A. Patients with unilateral lymphedema. B. Patients with bilateral lymphedema Effectiveness percentages are based on those who reported doing the exercises. The category "Done" constitutes the percentage of respondents who did the various exercises

Exercise types considered were low-impact and high-impact exercises, weightlifting, stretching, balance training, and flexibility training. The most frequently performed exercises were stretching and low-impact exercises. Results for patients with UELE(1) and for patients with UELE(2) are shown in Figure 1. Among UELE(1) patients, 78.1% did stretching and 70.5% did low-impact exercises. Of those who did these exercises, 78.0% found stretching to be somewhat or very effective and 85.1% found the low-impact exercise to be somewhat or very effective. Among UELE(2) patients, 80% did stretching and 66.7% did low-impact exercise. Of those who did these exercises, 75.0% found stretching to be somewhat or very effective and 90.0% found the low-impact exercise to be somewhat or very effective.

Preventative and Management Techniques

**Figure 2:**
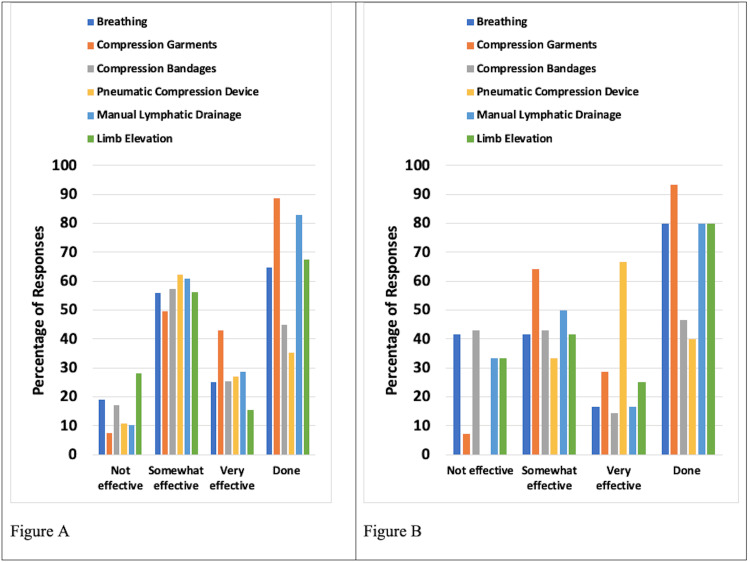
Preventative and management types and their reported effectiveness for patients with upper extremity lymphedema A. Patients with unilateral lymphedema. B. Patients with bilateral lymphedema Effectiveness percentages are based on those who reported using the methods. The category "Done" constitutes the percentage of respondents who used the various methods

The preventative and management techniques evaluated were compression garments, compression bandages, pneumatic compression devices, self-manual lymphatic drainage (MLD), and limb elevation. Among these, the most frequently used were compression bandages and manual lymphatic drainage. Results for patients with UELE(1) and for patients with UELE(2) are shown in Figure 2. Among UELE(1) patients, 44.8% used compression bandaging and 82.9% did MLD. Of those who did compression, 83.0% found it to be somewhat or very effective and 89.7% found MLD to be somewhat or very effective. Among UELE(2) patients, 46.7% did compression bandaging and 80.0% did MLD. Of those, only 57.1% found compression to be somewhat or very effective while 66.7% found MLD to be somewhat or very effective.

Dietary Approaches

**Figure 3:**
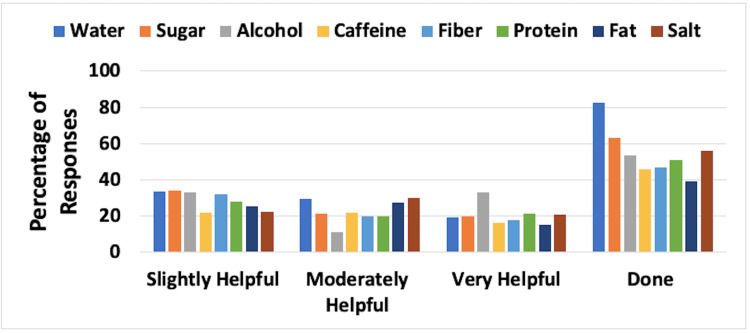
Dietary approaches and their reported effectiveness for patients with upper extremity lymphedema The figure is for combined unilateral and bilateral lymphedema patients. Effectiveness percentages are based on those who reported using the methods. The category “Done” constitutes the percentage of respondents who used the various methods. Approaches for water, fiber, and protein involved increasing consumption, while for the rest, the approach involved decreasing consumption

Dietary aspects evaluated were the frequency and effectiveness of increased water consumption, less sugar, less alcohol, less caffeine, increased fiber, increased protein, less fat, and less salt. Combined results for UELE(1) and UELE(2) are shown in Figure 3. The most frequently employed dietary change was increased water intake, with 82.5% of the 120 patients indicating they tried this approach. Of those, 48.7% indicated that it was moderately or very helpful. Alcohol reduction was the second most frequent change that was employed (53.3%), and of those patients, 43.9% believed it was moderately or very helpful.

Social Support Mechanisms Utilized

**Figure 4:**
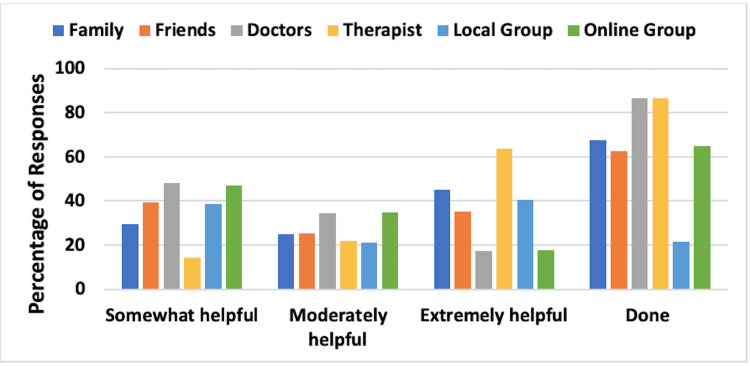
Social support mechanisms and their reported effectiveness for patients with upper extremity lymphedema The figure is for combined unilateral and bilateral lymphedema patients. Effectiveness percentages are based on those who reported using the mechanisms. The category “Done” constitutes the percentage of respondents who used the various support mechanisms

The aspects of social support evaluated were the frequency and effectiveness of support from family, friends, doctors, therapists, local support groups, and online support groups. Combined results for UELE(1) and UELE(2) are shown in Figure 4. The most frequently used support category involved the patient’s therapist, as indicated by 120 patients (86.7%). Of those, 85.7% reported that such support was moderately or extremely helpful. Support of the patient’s doctor was chosen by 86.7% of respondents, with 51.9% indicating that this was moderately or extremely helpful.

Patients with lower extremity lymphedema

As noted in Table 2, there were 280 patients with LE lymphedema.

Restrictions on Activities of Daily Living

As shown in Table 5, 37.4% of patients who had LELE(1) reported no or mild restrictions, 18.2% had severe or very severe restrictions, and the highest proportion of patients (44.4%) reported moderate restrictions. For patients with LELE(2), 23.8% indicated no or mild restrictions, but 40.8% reported severe or very severe restrictions.

**Table 5:** Reported restriction levels due to lower extremity lymphedematous condition

	Unilateral, %	Bilateral, %
No restriction	7.1	2.8
Mild	30.3	21.0
Moderate	44.4	35.4
Severe	15.2	30.9
Very severe	3.0	9.9

Exercise Methods Employed and Their Reported Effectiveness

**Figure 5:**
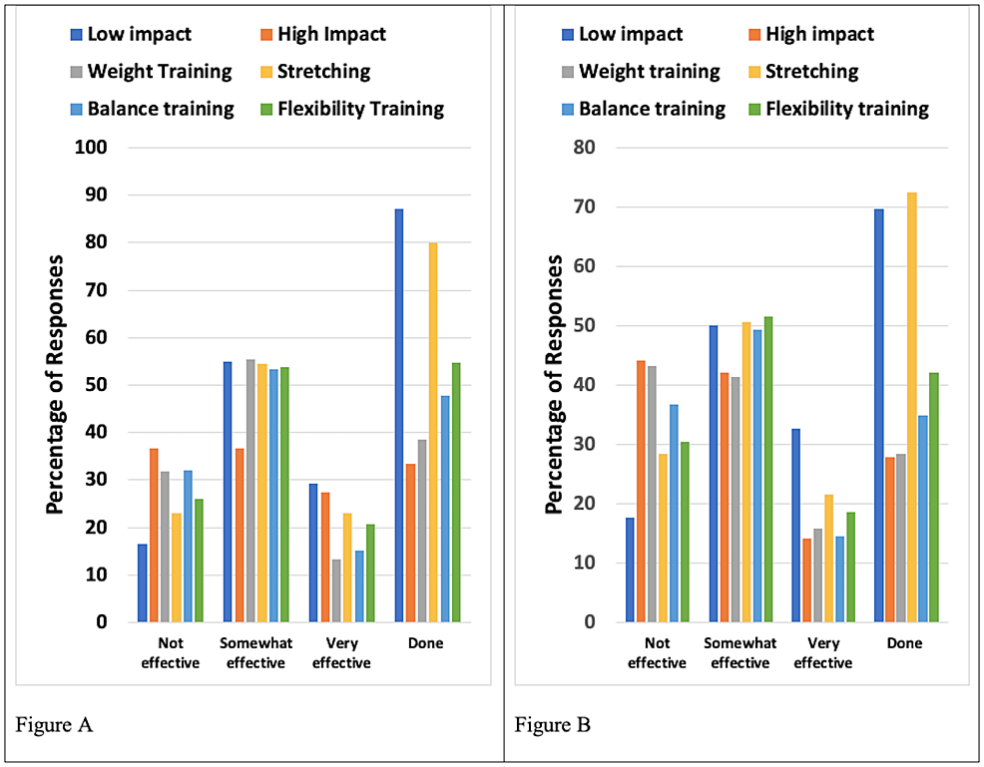
Exercise types and their reported effectiveness for patients with lower extremity lymphedema A. Patients with unilateral lymphedema. B. Patients with bilateral lymphedema Effectiveness percentages are based on those who reported doing the exercises. The category "Done" constitutes the percentage of respondents who did the various exercises

As with patients with UE lymphedema, patients with LE lymphedema also reported stretching and low-impact exercises as the most frequently employed exercise methods. Results for patients with LELE(1) and for LELE(2) are shown in Figure 5. In the LELE(1) group, 86.9% performed low-impact exercises, and 79.8% did stretching. Of those, 87.7% found the low-impact exercise to be somewhat or very effective and 77.2% found stretching to be somewhat or very effective. Among LELE(2) patients, 72.4% did stretching and 69.6% did low-impact exercise. Of those, 71.8% found stretching to be somewhat or very effective and 82.5% found the low-impact exercise to be somewhat or very effective.

Preventative and Management Techniques

**Figure 6:**
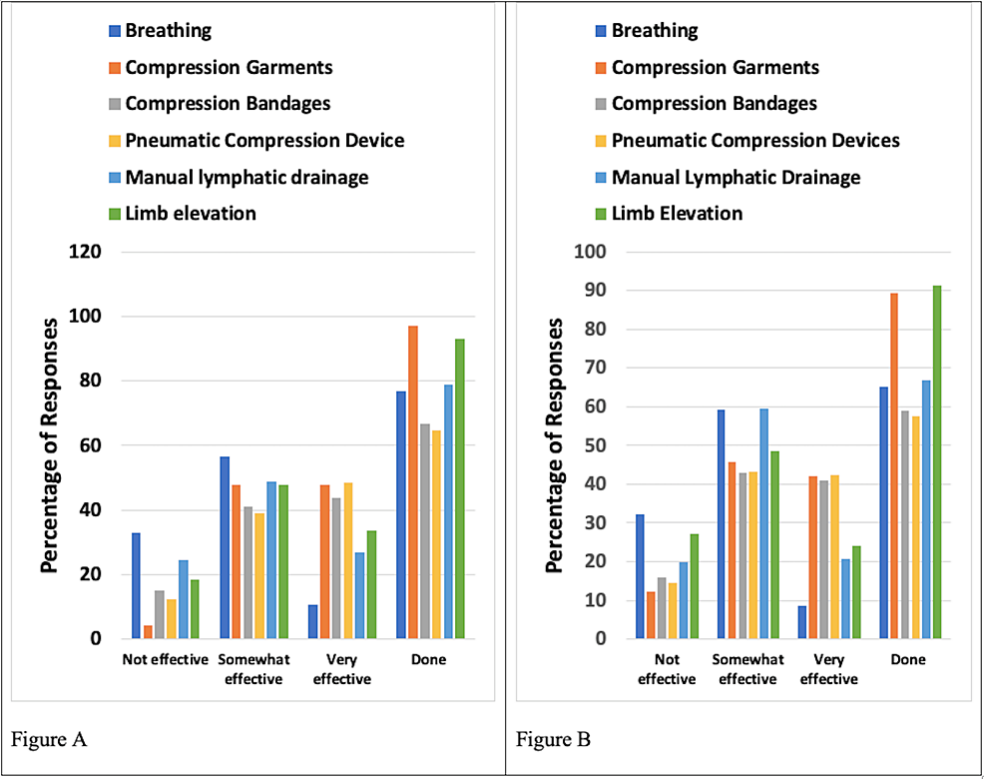
Preventative and management methods and their reported effectiveness for patients with lower extremity lymphedema A. Patients with unilateral lymphedema. B. Patients with bilateral lymphedema Effectiveness percentages are based on those who reported using the methods. The category "Done" constitutes the percentage of respondents who used the various methods

The most frequently used approaches were compression garments and limb elevation. Results for patients with LELE(1) and those for patients with LELE(2) are shown in Figure 6. Among LELE(1) patients, 97.0% used compression garments, and 92.9% practiced limb elevation. Of those who used compression garments, 95.8% found it to be somewhat or very effective. Of those who practiced limb elevation, 81.5% found it to be somewhat or very effective. Among UELE(2) patients, 89.5% used a compression garment, and of those, 87.7% indicated it was somewhat or very effective. Limb elevation was practiced by 91.2% of UELE(2) patients, with 72.7% of those indicating it was somewhat or very effective.

Dietary Approaches

**Figure 7:**
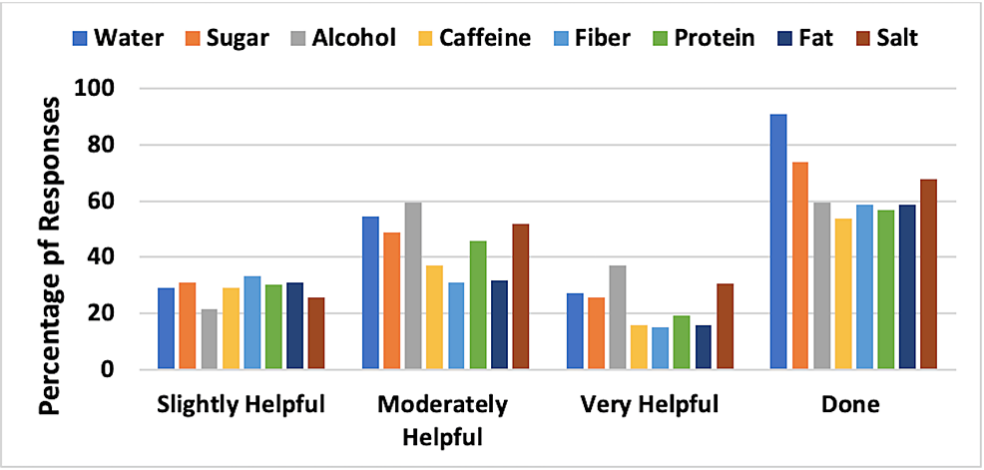
Dietary approaches and their reported effectiveness for patients with lower extremity lymphedema The figure is for combined unilateral and bilateral lymphedema patients. Effectiveness percentages are based on those who reported using the methods. The category “Done” constitutes the percentage of respondents who used the various methods. Approaches for water, fiber, and protein involved increasing consumption, while for the rest, the approach involved decreasing consumption

The combined results for LELE(1) and LELE(2) are shown in Figure 7. The most frequently employed dietary change was increased water intake with 91.1% of the 280 patients indicating that they tried this approach. Of those, 54.6% indicated that it was moderately or very helpful. Reduction of sugar intake was the second most frequent change; it was employed by 73.9% of respondents, and of those patients, 49.1% believed it was moderately or very helpful.

Social Support Mechanisms Utilized

**Figure 8:**
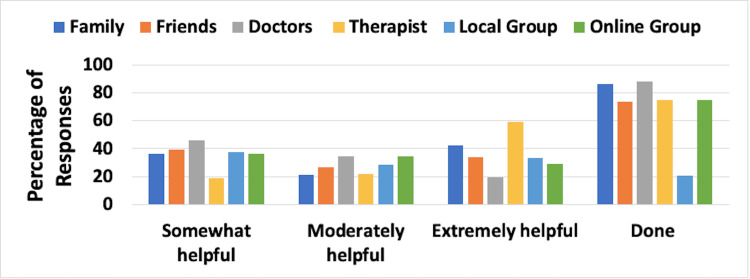
Social support mechanisms and their reported effectiveness for patients with lower extremity lymphedema The figure is for combined unilateral and bilateral lymphedema patients. Effectiveness percentages are based on those who reported using the mechanisms. The category “Done” constitutes the percentage of respondents who used the various support mechanisms

The combined results for LELE(1) and LELE(2) are shown in Figure 8. The most frequently used support category involved the patient’s doctor, as indicated by 280 patients (87.9%). Of those, 53.8% responded that this support was moderately or extremely helpful. Support of the patient’s family was a close second with 86.1% of patients reporting family as their source of support. Of those, 63.9% indicated that this support was moderately or extremely helpful.

## Discussion

The goal of this study was to devise guidelines for potential coping mechanisms for persons living with chronic lymphedema based on an investigation of the most effective reported coping strategies by current and previous lymphedema patients. Strategies for managing different areas of daily living, mobility, career, exercise, and travel as well as reported dietary approaches and social support mechanisms were assessed. Our findings can be employed to address the challenges the current lymphedema patients face every day.

Unilateral vs. bilateral considerations

Restrictions on activities of daily living among previous and current lymphedema patients were analyzed. Patients with UELE(1) are more likely to experience mild or moderate restrictions while patients with UELE(2) have a significantly higher probability of experiencing severe or very severe restrictions of daily living. Similarly, patients with LELE(1) mostly reported moderate restrictions while those with LELE(2) predominantly reported severe or very severe restrictions. The findings pertaining to the restrictive effect of both UE and LE lymphedema on patients’ quality of life are in line with the existing research, indicating that patients with limb lymphedema generally experienced negative impacts on their quality of life and functionality [3,4]. However, changes in arm volume appear to have a limited impact on patients' quality of life [5], and the importance of self-care and continuing follow-ups has been emphasized [6]. Our study also suggests a correlation between the increased severity of lymphedema and declining quality of life. In contrast, prior studies suggest that lymphedema severity did not appear to impact quality of life [7,8]. However, it is important to note that these studies analyzed the severity of lymphedema based on bioelectrical impedance measurement of limbs showing larger limb volume, as opposed to our study, which investigated the involvement of multiple limbs.

Exercise considerations

Different exercise modalities were investigated as a possible coping mechanism for lymphedema patients. The exercise categories analyzed included low-impact and high-impact exercises, weightlifting, stretching, balance training, and flexibility training methods. The most frequently performed exercises were stretching and low-impact aerobics. Patients with UELE(1) mostly used stretching and low-impact exercises with 78.0% of those respondents reporting stretching to be somewhat or very effective and 85.1% reporting low-impact exercises to be somewhat or very effective.

Among UELE(2) patients, most patients performed stretching, with low-impact exercise coming in second. Of those who did these exercises, 75.0% found stretching to be somewhat or very effective and 90.0% found the low-impact exercise to be somewhat or very effective. Similar to patients with UE lymphedema, patients with LE lymphedema also reported stretching and low-impact exercises as the most frequently performed exercises.

Among patients in the LELE(1) group, who mostly did stretching and low-impact exercises, 87.7% found the low-impact exercise to be somewhat or very effective and 77.2% found stretching to be somewhat or very effective. In the LELE(2) group, 71.8% found stretching to be somewhat or very effective and 82.5% found the low-impact exercise to be somewhat or very effective.

Our findings suggest that for both UE and LE lymphedema, stretching and low-impact aerobic exercise have a significant benefit in disease management. These findings are in line with the existing research, where studies attribute this to muscle contraction during light exercise leading to lymph drainage and protein absorption [9,10]. Furthermore, according to a meta-analysis of seven lymphedema studies, slowly progressive exercise is not associated with worsening of lymphedema symptoms [11].

Compression considerations

The preventative and management techniques evaluated were compression garments, compression bandages, pneumatic compression devices, self-MLD, and limb elevation. The most frequently used techniques among UE lymphedema respondents were compression bandages and MLD. Among UELE(1) patients, the majority did MLD and 89.7% of those respondents found it to be somewhat or very effective. Compression bandaging was used by less than half of the respondents, and of those who did compression, 83.0% found it to be somewhat or very effective. Similarly, among UELE(2) patients, the majority used MLD, and less than half used compression bandaging. Of those, only 66.7% found MLD to be somewhat or very effective and 57.1% found compression to be somewhat or very effective. Our findings are in line with evidence from prior studies that suggest that compression therapy significantly reduces limb volume, pain, and other symptoms of lymphoedema [12]. Our results also align with some studies reporting positive effects of MLD on lymphedema symptoms and quality of life compared with other treatments [13]. However, some other studies have reported no additional benefit from MLD [13].

For LE lymphedema, the most frequently used management approaches were compression garments and limb elevation. Among LELE(1) patients, nearly all used compression garments and practiced limb elevation. Of those who used compression garments, 95.8% found it to be somewhat or very effective. Of those who practiced limb elevation, 81.5% found it to be somewhat or very effective. Among LELE(2) patients, the majority used a compression garment, and of those, 87.7% indicated it was somewhat or very effective. Limb elevation was practiced in the majority of LELE(2) patients with 72.7% of those indicating that it was somewhat or very effective. Hence, our research indicates that MLD and bandage compression are the most effective management and preventative techniques among UE lymphedema patients with unilateral lymphedema showing significantly higher benefits compared to bilateral lymphedema. Moreover, patients with both unilateral and bilateral LE lymphedema reported similar benefits from compression garments and limb elevation. Lymphedema patients would benefit from using these management techniques to cope with their diagnoses.

Dietary considerations

The dietary aspects evaluated were the frequency and effectiveness of increased water intake, less sugar, less alcohol, less caffeine, increased fiber, increased protein, less fat, and less salt. The most frequently employed dietary change among UE lymphedema patients was increased water intake with a majority of the respondents indicating they tried this approach. Of those, approximately half indicated that it was moderately or very helpful. Similarly, the most frequently employed dietary change among LE lymphedema patients was increased water intake with a majority indicating they tried this approach. Of those, more than half indicated that it was moderately or very helpful. The survey did not further investigate the mechanisms whereby increasing the water intake was helpful. This may be a new finding that can be further explored.

Among respondents with UE lymphedema, reduction of alcohol intake was the second most frequent change employed, and of those that reduced alcohol intake, approximately half believed that it was moderately or very helpful. The exact impact of alcohol reduction on coping is unclear and is worthy of further investigation. Among respondents with LE lymphedema, reduction of sugar intake was the second most frequent change employed, as indicated by about three-fourths of respondents, and of those patients, approximately half believed that it was moderately or very helpful. Hence, our research suggests that increased water intake has a significant benefit in managing both UE and LE lymphedema. Decreasing sugar intake in UE lymphedema patients and decreasing alcohol intake in LE lymphedema patients showed similar results and may be beneficial coping mechanisms.

Social support considerations

The sources of social support utilized by patients were family, friends, doctors, therapists, local support groups, and online support groups, and the effectiveness of these support aspects was analyzed. The most frequently used support category among UE lymphedema patients was the patient’s therapist and doctor. The majority of those respondents reported the therapist's support to be moderately or extremely helpful while only half of them reported the same effectiveness for their doctor’s support. Among LE lymphedema patients, the most frequently used support category was the patient’s doctor with approximately half responding that this support was moderately or extremely helpful. Support of the patient’s family was a close second with the majority of patients reporting family as their source of support and about two-thirds of those patients indicating that this support was moderately or extremely helpful. Lymphedema diagnosis leads to a negative psychosocial impact on affected individuals [2]. A study has shown that lymphedema group-based education showed an improvement in quality of life among women with breast cancer-related lymphedema [14].

Study limitations

This study was confined to participants selected based on surveys distributed to online lymphedema support groups, and this limitation needs to be considered while interpreting the results as all lymphedema patients may not use or may not have access to online support groups.

## Conclusions

Based on the experiences of the lymphedema patients surveyed, the management of the condition is multifactorial and a one-size-fits-all strategy is not compatible with it. LE lymphedema was more common than the UE variant; but both groups reported engaging in stretching, low-impact exercise, manual lymphatic drainage, and compression bandages with similar satisfaction among all groups. Dietary changes were not commonly employed. Therapy, doctor, and family were the sources of support most commonly relied on with high satisfaction among both groups. Based on the survey results, there is still room for improvement in the management of lymphedema in terms of coping mechanisms. There are many unexplored coping mechanisms that could provide benefit to this patient group. Future research efforts in this field could include studies that employ specific strategies of interest to determine their effectiveness. The overall effectiveness of coping mechanisms was similar between upper and lower extremity groups although the impact on quality of life was greater for bilateral conditions. Our findings constitute a first step to providing information potentially useful to aid future lymphedema patients in finding the coping methods that work best for them.
